# Lipidic cubic-phase leflunomide nanoparticles (cubosomes) as a potential tool for breast cancer management

**DOI:** 10.1080/10717544.2022.2079770

**Published:** 2022-05-26

**Authors:** Mariam Zewail, Passent M. E. Gaafar, Mai M. Ali, Haidy Abbas

**Affiliations:** a Pharmaceutics Department, Faculty of Pharmacy, Damanhour University, Damanhour, Egypt; b Department of Pharmaceutics, Division of Pharmaceutical Sciences, College of Pharmacy, Arab Academy for Science, Technology and Maritime Transport, Alexandria, Egypt

**Keywords:** Cubosomes, leflunomide, passive targeting, cell uptake, antitumor, breast cancer

## Abstract

Despite the fact of availability of several treatments for breast cancer, most of them fail to attain the desired therapeutic response due to their poor bioavailability, high doses, non-selectivity and as a result systemic toxicity. Here in an attempt made to study the transdermal effect of leflunomide (LEF) against breast cancer. In order to improve the poor physicochemical properties of LEF, it was loaded into cubosomes. Cubosomes were prepared by the emulsification method. Colloidal characteristics of cubosomes including particle size, ζ-potential, entrapment efficiency, in-vitro release profile and ex-vivo permeation were studied. In addition, morphology, stability, cytotoxicity and cell uptake in MDA-MB-231 cell line were carried out for the selected cubosomal formulation. The selected LEF loaded cubosomal formulation showed a small particle size (168 ± 1.08) with narrow size distribution (PI 0.186 ± 0.125) and negative ζ potential (–25.5 ± 0.98). Its Entrapment efficiency (EE%) was 93.2% and showed sustained release profile that extended for 24 h. The selected formulation showed stability when stored at 25 °C for three months in terms of size and EE%. TEM images illustrated the cubic structure of the cubosome. Cell culture results revealed the superiority of LEF cubosomes compared to LEF suspension in their cytotoxic effects with an IC50 close to that of doxorubicin. Furthermore, LEF cell uptake was significantly higher for LEF cubosomes. This may be attributed to the effect of nano-encapsulation on enhancing drug pharmacological effects and uptake indicating the potential usefulness of LEF cubosomes for breast cancer management.

## 1. Introduction

The word ‘cancer’ refers to various malignancy cases that require medical intervention (Costa, Amorim et al., 2020). In the US, breast cancer accounts for 30% of all new cancer diagnoses in women and considered the leading cause of death among women aged 20 to 59 years (Siegel et al., 2019). Breast cancer is not linked only to an inherited genetic mutation but development and progression are more frequently connected with lifestyle, environmental and hormonal factors (Iacoviello et al., 2021) in addition to exposure to radiation that increase the risk of tumor genesis (Jain et al., 2020). Breast cancer is classified into four major molecular subtypes (Tong et al., 2018) and consequently, treatment protocols of breast cancer depend on its type and severity.

Breast cancer treatment requires multidisciplinary collaboration for operation interventions, systemic and radiation treatments (Tong et al., 2018). Although conventional treatments were used for decades, they mostly fail to attain the desired therapeutic response due to the poor physicochemical properties and bioavailability of anticancer drugs, required high therapeutic doses, drug non-selectivity which collectively result in therapeutic failure (Mehanna et al., 2020).

Transdermal drug delivery is a noninvasive route for systemic delivery of drugs across the skin layers. It has several merits compared to oral or parenteral routes as it can overcome gastrointestinal barriers, first pass metabolism in addition to its acceptability for patients (Nasr et al., 2020). Nanotechnology has numerous applications in diagnosing, monitoring, imaging, and delivering drugs to the tumor site (Vieira & Gamarra, 2016; Jain et al., 2020). Nanocarriers like polymeric nanoparticles (Jain et al., 2013; Li, Sun et al., 2015), liposomes (Ahmad et al., 2016), micelles (Guo et al., 2019), microspheres (Pal et al., 2019), dendrimers (Gupta et al., 2010; Chittasupho et al., 2017), solid lipid nanoparticles (Fontana et al., 2005; Acevedo-Morantes et al., 2013) and nanostructured lipid carriers (Sun et al., 2014) have been investigated over the past decade to increase the therapeutic efficacy of chemotherapeutic agents delivery to the targeted tumor site (Jain et al., 2020).

Liquid crystalline nanoparticles (LCNPs) or cubosomes are considered the non-lamellar analogue of liposomes that can penetrate skin and mucosa due to the similarity between their structure and the structure of the epithelial cell membrane and as a consequence the bioavailability of encapsulated drug in cubosomes is increased (Nasr et al., 2020). Like liposomes, in aqueous medium, cubosomes are self-assembled carriers but the lipid bilayer components in cubosomes are twisted resulting in the formation of 3D structures with continuous hydrophilic and hydrophobic regions (Luo et al., 2015). In cubosomes, the lipid matrix completely fills the inner portion of the nanocrystals and therefore cubosomes can offer a greater hydrophobic volume up to three times greater than liposomes meanwhile exposing only 60% of their surface to water with respect to liposomes (Jenni, Picci et al., 2020). As a consequence, cubosomes can increase the encapsulation efficiency of hydrophobic drugs in addition to their ability to encapsulate hydrophilic ones and vaccines (Luo et al., 2015).

Leflunomide (LEF) is an isoxazole derivative prodrug that was first approved for rheumatoid arthritis treatment at 1998. It showed promising results for its application as an anti-tumor agent. Upon administration, LEF is completely metabolized to its active metabolite teriflunomide (A771726) (Zhang & Chu, 2018). The prodrug activation was even confirmed in the skin upon topical administration (Bae & Park, 2016). LEF exerts its pharmacological anti-tumor effects by several mechanisms. It can act by inhibiting the *de novo* pyrimidine biosynthesis through suppression of mitochondrial dihydroorotatedehydrogenase (DHODH) enzyme which plays a vital role in cancer cells apoptosis through suppression of B and T cells (Sanders & Harisdangkul, 2002; Keen et al., 2013; Zhang & Chu, 2018; Zewail, 2021). Also, LEF can act as a tyrosine kinase inhibitor and was used for the treatment of several types of tumors (Pytel, Sliwinski et al., 2009). In addition, LEF showed selective inhibition of platelet-derived growth factor (PDGF) mediated phosphorylation. Signals through PDGF stimulate numerous functions such as cell growth, proliferation, and differentiation (Zhang & Chu, 2018). Furthermore, LEF can act as aryl hydrocarbon receptor (AhR) agonist by stimulating AhR which can inhibit cancer cells’ proliferation and act as a tumor suppressor in cancer animal models (O’Donnell et al., 2010). Furthermore, it was reported that LEF can inhibit stemness of cancer stem cells (White et al., 2011).

It was previously reported that LEF has anti-angiogenic and anti-proliferative effects against Ehrlich’s ascites carcinoma and that was attributed to its ability to down regulate of EGF and EGFR (Bahr et al., 2015; Zhang & Chu, 2018). This is along with the results previously reported by Huang et al. (2015) about the anticancer effect of LEF metabolite against triple negative breast cancer cells (Huang et al., 2015).

Unfortunately, LEF possess several gastrointestinal side effects including stomatitis, diarrhea, colitis and esophagitis, besides to its systemic side effects on the skin, liver and lungs (El-Setouhy et al., 2015; Zewail et al., 2019).

The aim of the current study is the investigation of the anti-cancerous effects of LEF against breast cancer. In order to maximize LEF therapeutic effects and decrease its side effects, LEF loaded cubosomes for transdermal drug delivery were prepared. The in-vitro characteristics of LEF loaded cubosomes in addition to ex-vivo permeation of cubosomes were investigated in addition to studying cubosomes’ cytotoxicity and uptake by breast cancer cell line (MDA-MB-231).

## 2. Materials

Leflunomide was purchased from Qingdao Franken Biochem Co. (Qingdao, China). Peceol^®^ (glyceryl monooleate) was kindly provided of by Gattefosse (Lyon, France). Poloxamer^®^ 407 was purchased from BASF chemical company (Ludwigshafen, Germany). Oleic acid was bought from El-Nasr Pharmaceutical (Cairo, Egypt). Fetal bovine serum (FBS), (Dulbecco's Modified Eagle Medium) (DMEM), trypsin/EDTA, and Penicillin streptomycin, were purchased from Lonza company, Switzerland. All other chemicals were of analytical grade.

## 3. Methods

### 3.1. Preparation of LEF loaded cubosomes

LEF loaded cubosomes were prepared by the emulsification of a mixture of glyceryl monooleate (GMO) by the surfactant in water as described by Badie & Abbas (2018) and Esposito et al. (2005). The composition of the formulations is shown in Table 1.

**Table 1.** Composition of LEF loaded cubosomes.

Formulation	(%w/w)			
	LEF	OA	POL	GMO
**F1**	2	0.5	0.5	9
**F2**	2	0.5	0.5	4.5
**F3**	2	0	0.5	9
**F4**	2	0	0.5	4.5

Briefly, the dispersed phase consisting of Oleic acid (OA), Poloxamer 407 (POL) and GMO were melted in a thermostatically controlled water bath (GFL, type 1083, Gmbh & Co., Germany) at 70 °C, then LEF was solubilized in the mixture with the help of a vortex mixer for 5 sec. The resultant mixture was added dropwise to a preheated aqueous phase at 70 °C while stirring followed by emulsification using a rotor-stator homogenizer (T25 digital ULTRA-TURRAX VR, Germany) at 10,000 rpm for 5 min. The final cubosomal dispersion was left to cool at room temperature and maintained at room temperature for further investigations.

### 3.2. Particle size analysis and zeta potential measurements

Cubosomal formulations were subjected to particle size, PDI and ζ potential analysis by using Malvern zeta sizer Nano ZS, Malvern Instruments, (Malvern, UK). Formulations were suitably diluted with deionized water. Samples for particle size measurements were placed in square glass cuvettes, while those for *ζ* potential were placed in clear disposable zeta cells. Measurements were repeated in triplicates and the values were expressed as mean ± SD.

### 3.3. Entrapment efficiency percentage (% EE)

The amount of LEF encapsulated in the prepared cubosomes was determined by a reported (El-Setouhy et al., 2015; Zewail et al., 2019; Zewail, 2021) indirect method. Briefly, a 1-mL aliquot LEF loaded cubosomal suspension was transferred into a centrifuge tube fitted with an ultrafilter (Vivaspin 6VR, Sartorius, MWCO 10 kDa) and then centrifuged at 4000 rpm for 1 h at 4 °C (Sigma Laboratory Refrigerated Centrifuge, Model 3 K-3o, Germany). The amount of free/unencapsulated LEF in the filtrate was determined spectrophotometrically at 263 nm and the EE % was determined according to the following equation:

$$\text{EE \%}=\frac{\text{Total amount ofLEF}-\text{ Amount of unencapsulted LEF}}{\text{Total amount of LEF}}\times 100$$

### 3.4. X Ray diffraction

X-ray diffraction analysis for LEF and different cubosomal formulations was performed using Philips-PW-1050 X-ray diffractometer at voltage of 20 mA and current of 40 kV using Ni filter and Cu Ka radiation (Holland).

### 3.5. In-vitro *drug release and release kinetics*

Release of LEF from suspension and different cubosomal formualtions was investigated by dialysis technique. Appropriate volumes of LEF suspension and LEF loaded cubosomes (equivalent to 500 µg LEF) were put in dialysis bags, which were then immersed in flasks containing PBS (75 mL, pH 7.4 to ensure sink conditions). The flasks were incubated at 37 ± 0.2 °C in a shaking water bath moving at 100 rpm. At different time intervals (1,2,3,4,6,8 and 24 hours), samples were withdrawn and replaced with fresh PBS. LEF concentration was determined by the first derivative of the UV spectrum at 248 nm. All measurements were carried out in triplicates and the values were expressed as mean ± SD.

To analyze the release kinetics of LEF loaded cubosomes, release data were fitted into zero-order, first order and Higuchi model using DD solver software.

### 3.6. Ex vivo skin permeation test

The skin of newly born Albino female mice (age 6 days or younger) was chosen as a model membrane. After mice sacrificing, the skin was carefully excised, washed three times with PBS (pH 7.4) and then examined macroscopically for any defect prior to the experiment.

Transdermal permeation of LEF loaded cubosomes was conducted using a modified Franz diffusion cell assembly under non-occlusive conditions (Mohyeldin et al., 2016). A Franz cell with a contact area of 3.14 cm^2^ and a receptor volume of 8.5 mL was employed in the permeation study.

Skin samples were placed between the donor and receptor compartments of the diffusion cell with the stratum corneum side facing the donor compartment. The receptor compartment was filled with 8.5 mL of 1% ethanolic/PBS pH 7.4 to provide sink conditions. The diffusion cells were maintained at 32 ± 0.5 °C and continuously shaken at 100 rpm in a thermostatically shaking controlled water bath. After 15 min equilibration, LEF suspension or the selected cubosomal formulation (equivalent to 500 micrograms LEF) were added in the donor compartment. Samples were withdrawn from the receptor compartment at predetermined time intervals (2, 4, 6, 8, 12, 24, 32 and 48 hours) and were compensated with an equal volume of fresh buffer. Each sample was filtered through 0.22 μm syringe filter and amount of LEF permeated was quantified spectrophotometrically at 248 nm.

### 3.7. Permeation data analysis

The in vitro skin permeation data obtained was graphically plotted as the cumulative amount of LEF (μg/cm^2^) permeated into the receptor phase as a function of time. The slope of the straight linear portion of this plot yielded the values of flux Jss (μg.cm^−2^h^−1^) (Can, Erdal et al., 2013; Abd El Azim et al., 2015).

$$\mathrm{Jss}=\frac{dQ/dt}{A}$$

Where, Jss is the steady-state flux (μg.cm^−2^h^−1^); dQ/dt is the permeation rate (mg cm^−2^); A is the active diffusion area (cm^2^).

The permeability coefficient (*P)* was calculated using the relation derived from Fick’s first law of diffusion as follows:

$$P=\frac{\mathrm{Jss}}{Cd}$$

Where, *P* is the permeability coefficient (cm.s^–1^) and Cd is the concentration of drug in the donor compartment (μg/mL).

### 3.8. Morphological examination

Morphology of the selected cubosomal formulation was investigated using transmission electron microscopy, TEM, (JEM-100 CX, JEOL, Japan) at 80 kV. The sample was suitably diluted with distilled water and sonicated for 5 min. Then, a drop of the diluted dispersion was placed on a copper-coated grid followed by staining with phosphotungistic acid solution and air drying.

### 3.9. Storage stability

Stability of selected LEF loaded cubosomal formulation over three months at 25 ± 2 °C was tested through the measurement of their particle size, PDI and zeta potential and % EE.

### 3.10. Cytotoxicity against breast cancer cell line (MDA-MB-231)

#### 3.10.1. Cells

Cytotoxicity test was determined in Breast Cancer cell line (MDA-MB-231) (Nawah-Scientific, Cairo, Egypt). Cells were grown in DMEM medium supplemented with 10% FBS, 100 units/mL of penicillin, and 100 mg/mL of streptomycin. Cultures were maintained in a humidified atmosphere with 5% CO_2_ at 37 °C.

#### 3.10.2. Samples

Each sample was pre-solubilized in dimethylsulfoxide (DMSO) at 37 °C to give a stock solution. Serial ten-fold dilutions were made working concentrations of 10000, 1000, 100, 10, 1 …etc. (μg/mL).

#### 3.10.3. MTT assay

Briefly, Confluent monolayers of MDA-MB-231 cells were grown in 96 well-microtitre plates for 24 h. Cells were incubated with various concentrations of the test samples in triplicate at 37 °C in a CO_2_ environment for 48 h.

After that, gently added 20 μL MTT (5 mg/mL) to each well and incubated at 37 °C for 4 hrs. Then carefully remove media and add 150 μl MTT solvent. Cover with tinfoil and agitate cells on orbital shaker for 15 min. Finally, the OD was measured at 570 nm in microplate reader (BMGLABTECH^®^FLUOstar Omega, Germany).

### 3.11. Cell uptake

MDA-MB-231 cell line was used to study the cellular uptake of the provided LEF and the selected LEF loaded cubosomal formulation. On the first day, the cells were seeded in T25 flask. On the second day, the media were removed, and media supplemented with predetermined drug concentration were added to cells. A confluent T25 flask 0.7 × 106 of MDA-MB-231 cells was harvested after 6 and 24 hours of incubation. The pellets were collected at each time interval were subjected to lysis according to the method previously reported by Srisongkram et al. (Srisongkram & Weerapreeyakul, 2019). Sample preparation for HPLC analysis was carried out as follows: 0.5 mL methanol was added to each sample then sonicated for 15 min. This step was followed by centrifugation for 10 min at 4500 rpm and filtration using 0.22 μm Nylon syringe filter.

Amount of LEF taken by the cells were quantified using HPLC (Waters 2690 Alliance HPLC system equipped with a Waters 996 photodiode array detector). The Mobile phase consisted of (50%:50%) acetonitrile: phosphate buffer (0.02 M) pH was adjusted to 2.6 with orthophosphoric acid. Flow rate was 1 mL/min and the measurements were carried out at 260 nm (Zewail et al., 2019).

### 3.12. Statistical analysis

Data are presented as Mean ± SD and data analysis was performed using Graph Pad Prism 7 software.

## 4. Results and discussion

### 4.1. Preparation of LEF loaded cubosomes

There are limited studies on the preparation of LEF loaded nanocarriers. To our knowledge, this is the first study reporting the preparation of LEF loaded cubosomes, testing their effect on breast carcinoma cells and comparing this effect with drug suspension. LEF is a hydrophobic drug belonging to biopharmaceutical class II (El-Sayyad et al., 2017; Zewail et al., 2019) and therefore the choice of cubosomes was based on its several merits on loading hydrophobic drugs (Mehanna et al., 2020).

Cubosomes were prepared by top bottom method using GMO as a cubic phase precursor lipid. POL was used as a polymeric stabilizer where the hydrophobic polypropylene oxide portion adsorb onto the surface of the particles, while the hydrophilic polyethylene oxide portion probably extends out into the aqueous media providing steric hindrance (Gaballa et al., 2020; Mehanna et al., 2020). The GRAS listed unsaturated fatty acid OA was used for its potential as a penetration enhancer for transdermal drug delivery (El-Masry et al., 2018).

### 4.2. Colloidal characteristics of LEF loaded cubosomes

Various physicochemical characteristics of the prepared LEF loaded cubosomes are summarized in Table 2. Particle size of different formulations ranged from 168 (F4) to 215 nm (F1).

**Table 2.** Colloidal characteristics and % EE of different LEF loaded cubosomes.

Formulation	F1	F2	F3	F4	F4 (3 months)
Particle size (nm)	215 ± 1.58	175 ± 1.45	194 ± 2.15	168 ± 1.08	169 ± 1.74
PDI	0.406 ± 0.85	0.245 ± 0.75	0.325 ± 0.45	0.186 ± 0.12	0.244 ± 0.20
Zeta potential (mV)	–35.5 ± 1.60	–31.5 ± 1.47	–27.5 ± 1.25	–25.5 ± 0.98	–26.3 ± 1.18
EE%	89.6 ± 1.58	85.3 ± 1.15	87.3 ± 1.08	93.6 ± 1.96	92.8 ± 1.45

**Table 3.** Release kinetics of different LEF loaded cubosomes.

Release model	R^2^				
	LEF suspension	F1	F2	F3	F4
Zero order	0.579	0.546	0.586	0.452	0.770
First order	0.677	0.886	0.961	0.959	0.839
Higuchi	0.920	0.959	0.959	0.923	0.941

**Figure 1.**
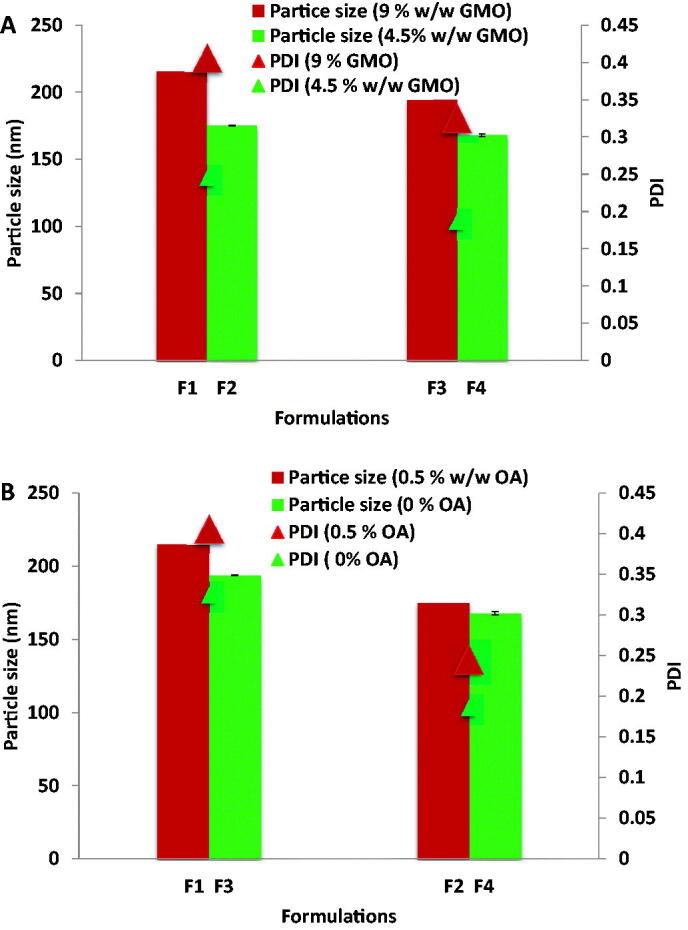
(A) Effect of GMO concentration on particle size and PDI of cubosomes. (B) Effect of OA concentration on particle size and PDI of cubosomes.

Increasing GMO concentration resulted in a significant increase in particle size and PDI of cubosomes (student *t*-test (*p* < .05)) as shown in Figure 1. This may be attributed to the effect of higher GMO concentration on aqueous phase uniform dispersion resulting in heterogeneous particle size distribution (Badie & Abbas, 2018). Also, Figure 1(B) shows that increasing OA concentration resulted in a significant (*p* < .05) increase in particle size and PDI values of cubosomes upon applying student *t*-test.

Zeta potential is an important indication of the stability, where formulations with zeta potential values around ± 30 mV are considered stable (Ahmed, Kassem et al., 2020) due to repulsive forces that prevent particles coalescence and aggregation (Rahman, Rasedee et al., 2013; Zewail et al., 2019; Ahmed, Kassem et al., 2020). All the prepared cubosomal formulations carried high negative surface charge that ranged from –25.5 (F4) to –35.5 mV (F1) which can indicate their physical stability. It is interesting to note that the ζ potential was found to positively correlate with OA in the cubosomes. Formulations F1 and F2 containing OA showed significantly higher ζ potential than formulations F3 and F4 where OA was not added. However, the change in the free OA content of GMO did not significantly affect the ζ potential in cubosomal formulations F3 and F4.

The negative surface charge on LEF loaded cubosomes may result from the ionization of the carboxylic group of free OA added or present in GMO and adsorbed onto cubosomes’ surface or it may be attributed to the presence of polarized water layer that surrounds the outer cubosomes surface (Badie & Abbas, 2018). This negative ζ potential may be considered beneficial as it aids in increasing the cubosomes contact time by electrostatic interactions between negatively charged cubosomes and positively charged skin surface (El-Masry et al., 2018).

### 4.2. Entrapment efficiency (EE %)

All LEF loaded cubosomes showed high EE% that ranged from 85.3 (F2) to 93.6% (F4) as illustrated in Table 2. This might be attributed to the lipophilic nature of LEF that enables its successful incorporation in the hydrophobic domain of the cubic phase bilayer. This is in agreement with several studies that reported the high EE % of lipophilic drugs in cubosomes (Lai et al., 2009; Nazaruk et al., 2017). Decreasing GMO concentration didn’t significantly affect the entrapment efficiency due to presence of sufficient amount of GMO to entrap the used drug concentration. This is in accordance with reported data where, the entrapment efficiency of GMO cubosomes was not affected by GMO concentration (Hashem et al., 2018) or was not affected at GMO concentrations above 5% (Nasr et al., 2021).

Oleic acid was selected in the cubosomal formulation for its merits as a safe and potential penetration enhancer. Also, Oleic acid is reported (Salentinig et al., 2010) to increase the critical packing parameter of SAA and lipids in aggregates which may result in structural transitions from bicontinuous cubosomes through hexosomes and micellar cubosomes to emulsified microemulsions with increasing oleic acid concentration. Although the concentration of oleic acid in the present work is low (0.5%), it may have affected the bicontinuous structure of the prepared cubosomes reflected in lowering LEF entrapment potential. This ‘salting out’ effect of Oleic acid is more evident when GMO concentration decreased from 9% to 4.5% where %EE showed its minimum value (85.3 ± 1.15).

### 4.3. X Ray diffraction

**Figure 2.**
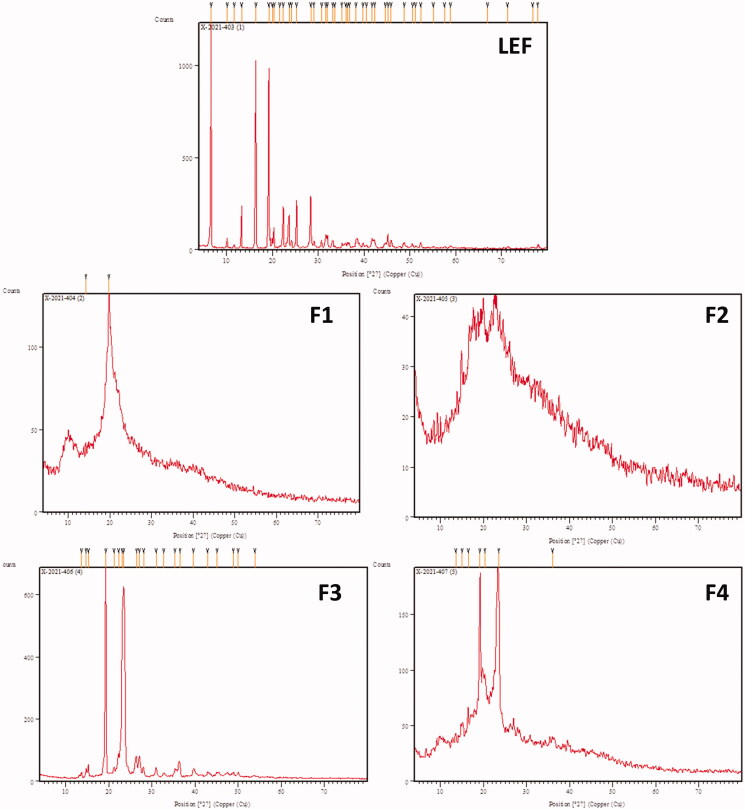
X ray diffraction of LEF and different cubosomal formulations.

X ray diffraction of LEF and different cubosomal formulations are illustrated in Figure 2. LEF diffraction showed specific sharp peaks with high intensity indicating the crystalline nature of LEF. On the other hand, LEF loaded cubosomes did not show peaks of high intensity indicating the presence of LEF in the amorphous state in the bicontinous structure of cubosomes. These findings are in agreement with previously reported results (Salah et al., 2017).

### 4.4. In vitro *drug release and release kinetics*

**Figure 3.**
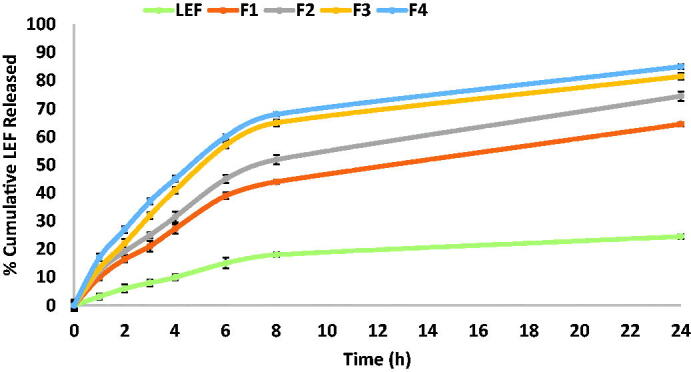
Cumulative percentage of LEF released from LEF loaded cubosomes using dialysis bag method.

Solubility of LEF was conducted in PBS (pH 7.4) and was found to be 2.67 mg/100 mL ensuring sink conditions for the release study. All LEF loaded cubosomes showed biphasic release pattern with an initial burst followed by sustained LEF release for 48 h (Figure 3(A)) in agreement with previously reported data (Dian et al., 2013). The initial burst effect may be attributed the drug adsorbed on the cubosome surface while the sustained release behavior may be attributed to the drug entrapped inside the cubosomes.

Increasing GMO and/or OA concentration resulted in decreasing drug release rate from cubosomes. Increasing GMO concentration increases matrix viscosity and this will in turn retard drug diffusion from the lipid bilayer to the aqueous release medium and eventually drug release rate is slowed down (Badie & Abbas, 2018). Also, formulations composed 0.5% OA like F2 showed slower release compared to F4 which does not contain OA. This may be attributed as mentioned above to the adsorption of OA at the cubosomal surface which may decrease the diffusion rate of the release medium and the drug.Slower release of LEF suspension in comparison with LEF cubosomes may be attributed to the effect of nanoencapsulation on increasing drug solubility and hence higher amount of drug are released from nanoformulation in comparison with drug suspension (Elmowafy & Al-Sanea, 2021). Our findings are consistent with the results reported by Ribeiro et al. (2012), who demonstrated that drug encapsulation could overcome solubility problems for poorly water-soluble drugs. As a result, the drug concentration gradient increases and releases a much higher drug concentration (Ribeiro et al., 2012).These findings are a long with the results reported by Abbas et al. (2021), who reported that herbal colloidal carriers loaded with curcumin and resveratrol demonstrated higher release percentages of both drugs compared with their corresponding drug suspensions (Abbas et al., 2021).

Several factors affect drug release kinetics from cubosomes such as drug physicochemical characteristics, interactions between the drug and the lipid domain, water content and drug loading percentage (Guo et al., 2010). The release kinetics of LEF suspension and different cubosomal formulations were fitted to zero, first and Higuchi model and their correlation coefficients (R^2^) were listed in Table 3. Results showed that Higuchi’s model showed the highest R^2^ in all formulations suggesting LEF release from cubosomes occurred by diffusion mechanism. This in turn supports the slowest release results obtained by the F1 cubosomal formulation having the highest % lipids due to hindered diffusion of the release medium to the center of cubosome and hindered drug diffusion outside the cubosome.

### 4.5. Ex-vivo *skin permeation test*

**Figure 4.**
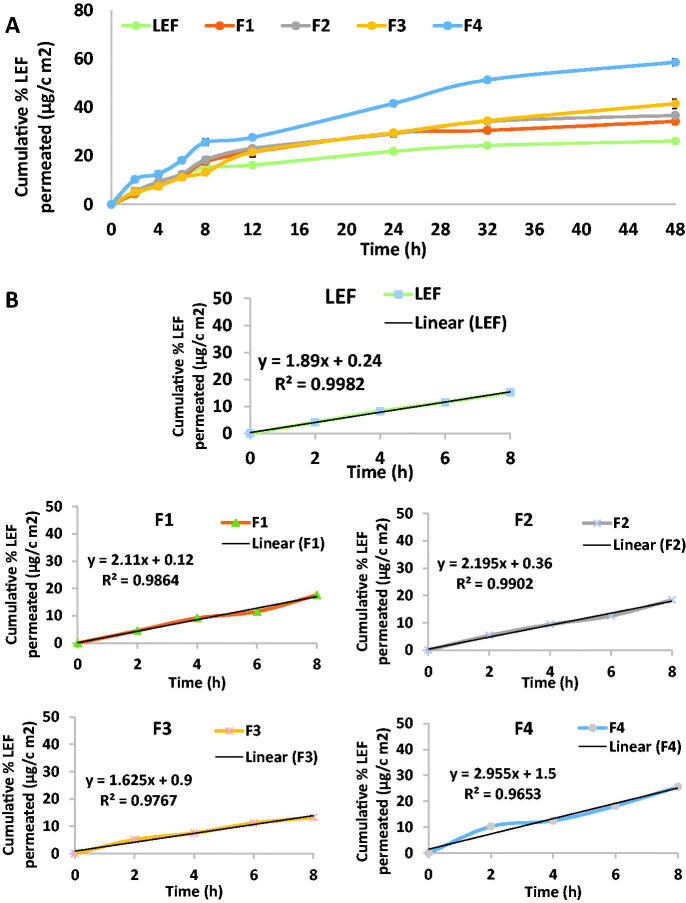
(A) Cumulative percentage LEF permeated through skin in *ex vivo* permeation test. (B) Linear correlation between cumulative percentage LEF permeated in *ex-vivo* for LEF suspension and different cubosomal formulation within the first 8 h of the experiment.

Ex-vivo permeation test was conducted to preliminarily predict the in-vivo behavior of LEF loaded cubosomes. The choice of hairless mouse skin is based on its reported similarity to the human skin structure (Chantasart, Li et al., 2004; Badie & Abbas, 2018). Solubility study of LEF was conducted in in 1% ethanolic/PBS pH 7.4 and was found to be 23.5 mg/100 mL. Thus, sink conditions was ensured during the permeation study. Cumulative LEF permeated from different LEF loaded cubosomes in comparison to LEF suspension after 8 hrs are illustrated in Figure 4(A). The curves showed a Fickian behavior where a linear correlation (R^2^ values ranged from 0.9653 to 0.9982) between cumulative LEF permeated in relation to time during the first 8 h of the experiment (Figure 4(B)). Formulation (F4) presented the highest permeation result which correlates with its highest release results.

At 48 hours, different cubosomal formulations showed the highest cumulative LEF permeated (34.2, 36.7, 41.5 and 58.6 μg/cm^2^ for formulations F1 to F4 respectively) compared to LEF suspension (26.1 μg/cm^2^).

These findings are in agreement with the previously reported results that demonstrated the ability of cubosomes to enhance transdermal drug delivery compared to drug suspension (Peng et al., 2015; Badie & Abbas, 2018; Nasr et al., 2020). In addition, F4 showed the highest cumulative percentage LEF permeated compared to other formulations in accordance with the drug release result (Figure 4(A)).

As Table 4 illustrates, F4 had the highest flux (2.995 μg.cm^−2^h^−1^) and permeability coefficient (0.0059 cm.s^–1^) compared with the other cubosomal formulations. All formulations except F3 had higher flux and permeation coefficient than LEF suspension.

**Table 4.** Permeation data analysis.

Formulation	Flux (μg.cm^–2^h^–1^)	Permeability coefficient (cm.s^–1^)
LEF	1.89	0.0037
F1	2.11	0.0042
F2	2.195	0.0043
F3	1.625	0.0032
F4	2.995	0.0059

### 4.6. In vitro *release and ex-vivo permeation co-relation*

Seeking to assess possible relation between in vitro release data and ex-vivo skin permeation data generated for different cubosomal formulations and LEF suspension, the percent cumulative amount permeated ex vivo across skin was plotted against the percent cumulative amount LEF released in vitro. Regression analysis was performed as Figure 4 illustrates. LEF suspension and LEF cubosomes showed good correlation between cumulative percentage drug released and cumulative drug permeated. F1 and F2 had the highest correlation coefficient R^2^ values were 0.984 and 0.983, respectively. Slope value of LEF suspension close to unity indicates comparable increments in percent drug released and permeated (Abd El Azim et al., 2015). Slope values of LEF cubosomes smaller than unity indicates that the amount of drug released is higher compared with the amount of drug permeated suggesting that there might be an interaction between LEF cubosomal formulations and the skin domains (stratum corneum) resulting in drug accumulation or formation of drug depot of the drug within the stratum corneum (Esposito et al., 2005; El-Enin & Al-Shanbari, 2018; Omar et al., 2019).

**Figure 5.**
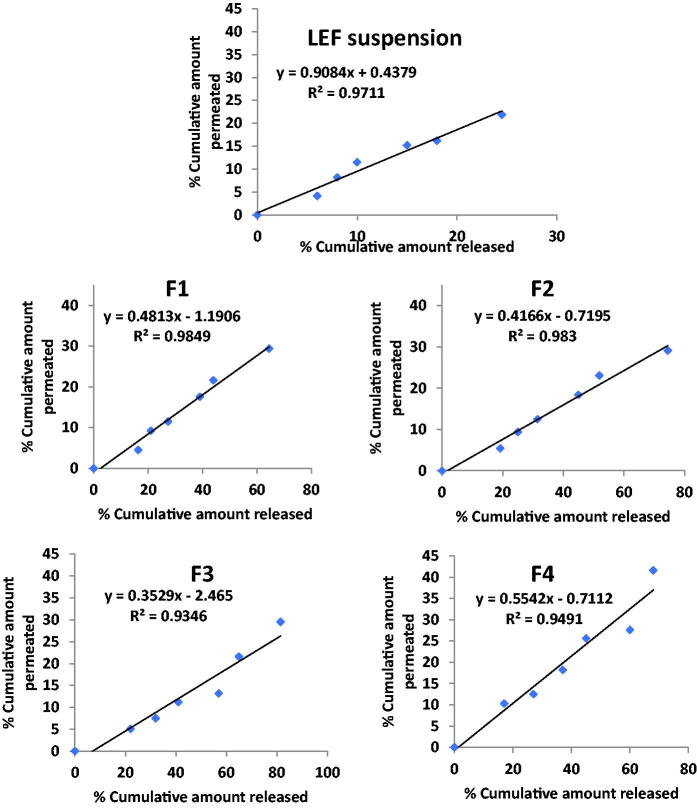
Correlation between percentage released and percentage permeated in different cubosomal formulations and LEF suspension.

From all the above results, LEF loaded cubosome (F4) was selected for further studies as it possess; the most uniform and small suitable particle size (168 nm), PDI (0.186), the highest EE% (93.6%), a suitable negative zeta potential (–25.5 mV) in addition to the highest percentage LEF released from cubosomes and permeated in ex-vivo permeation study (Figure 5).

### 4.7. Morphological examination

**Figure 6.**
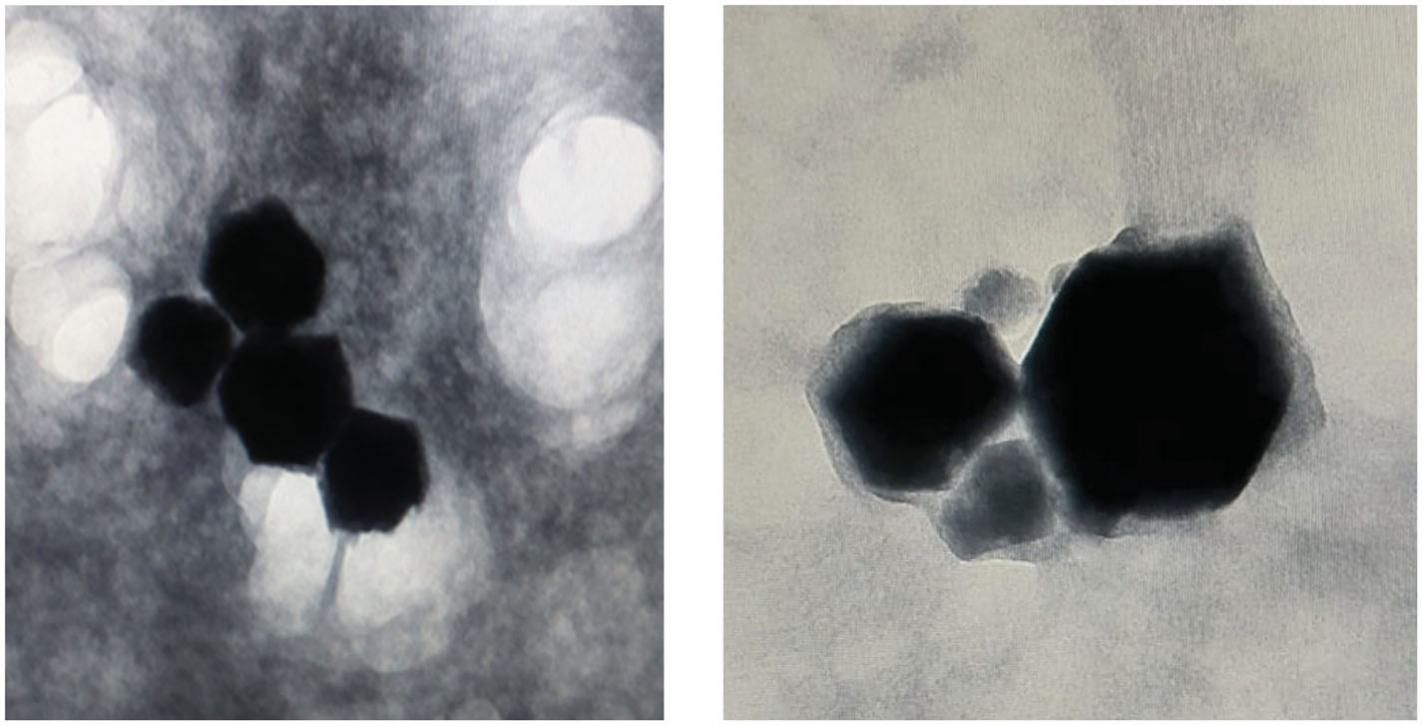
TEM micrographs of selected formulation of LEF loaded cubosomes (F4).

The morphological structure of the selected LEF loaded cubosomes revealed that they had a clear well-ordered cubic structure as shown in Figure 6. Absence of drug crystals indicates optimum drug entrapment within the cubosomes.

### 4.8. Storage stability

As seen from the results in Table 2, storage of the selected formulation (F4) at 25 °C ± 2 for three months did not significantly affect their physical attributes including particle size, PDI, zeta potential and EE %. This is in agreement with the previously reported results that indicated cubosomes stability over 3 month storage period (Elgindy et al., 2016; Mehanna et al., 2020).

### 4.9. Cytotoxicity assay

**Figure 7.**
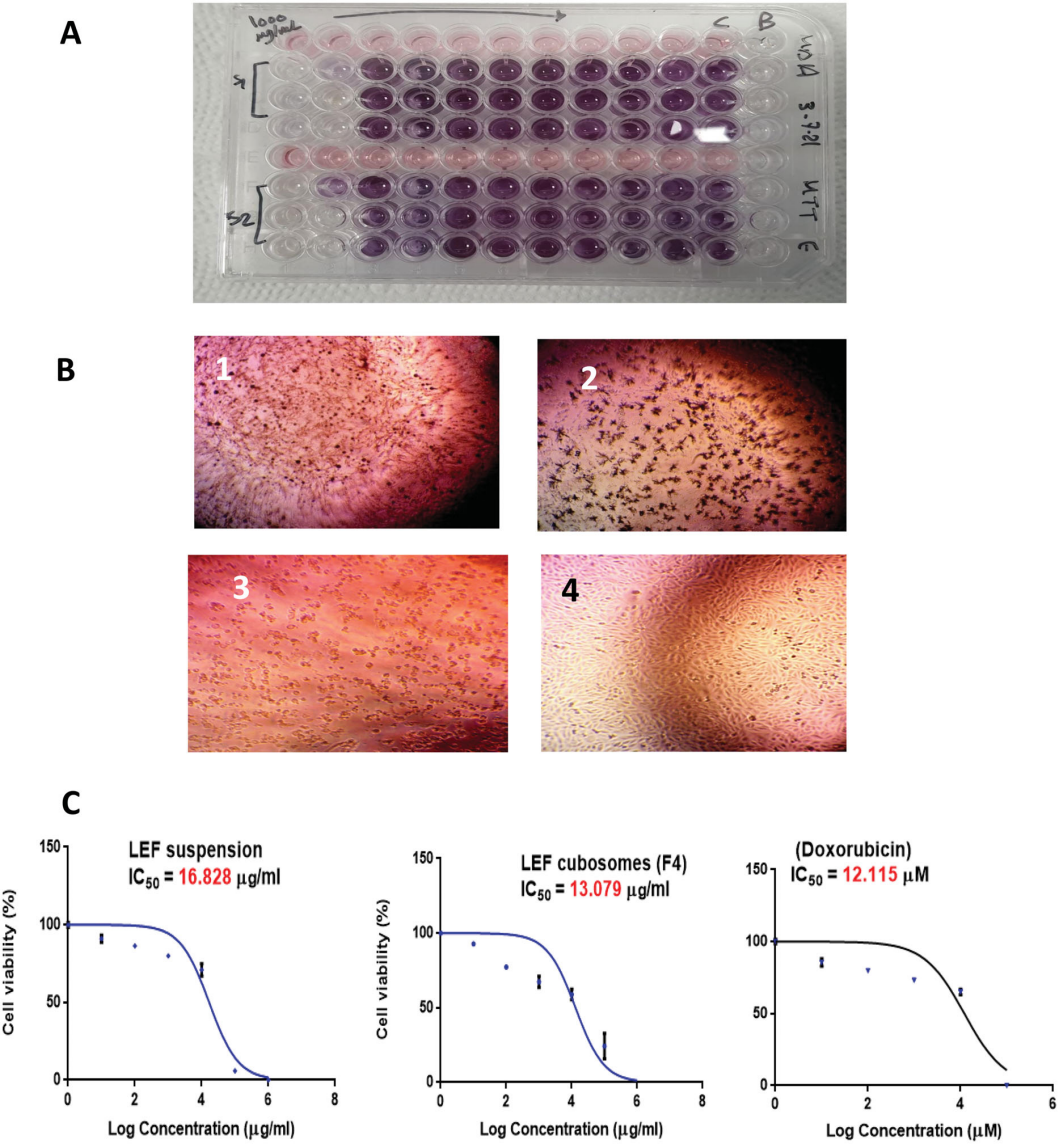
(A) MTT assay. (B) Morphological examination of cell line after treatment with 100 µg/mL of (1) LEF suspension, (2) LEF cubosomes (F4) and (3) Positive control (Doxorubicin). (4) MDA-MB-231 cells before treatment. (C) Percentage Cell viability of: LEF suspension, LEF cubosomes (F4) and positive control (doxorubicin).

In vitro cytotoxicity of LEF suspension, LEF cubosomes and Doxorubicin (Dox; positive control) was measured using the popular colorimetric MTT assay method. As shown in Figure 6(A), LEF suspension, LEF cubosomes and Dox displayed concentration-dependent cytotoxicity (Figure 7).

The IC50 values of free LEF, LEF cubosomes and Dox were 16.828 ± 0.093, 13.079 ± 0.1431 and 12.115 ± 0.132 μg/mL, respectively. LEF suspension was found to be cytotoxic to MDA-MB-231 in accordance with above mentioned data. LEF cubosomes reduced the viability of MDA-MB-231 significantly compared to LEF suspension upon data analysis with Two WAY ANOVA with *p* < .0001. This result indicates better delivery of the drug LEF from cubosomes compared to the suspension. The superior cytotoxicity of LEF cubosomes compared with LEF suspension is in agreement with the previously reported results of Mehanna et al. (2020) who confirmed the superiority of drug loaded cubosomes over free drug in different breast cancer cell lines. This improvement may be explained by the physicochemical characterization results, where the drug loaded cubosomes encapsulated the drug in an amorphous form in comparison to the crystalline form in the suspension formulation, showing a faster release profile of the drug which was reflected in its superior activity.

### 4.10. Cell uptake

**Figure 8.**
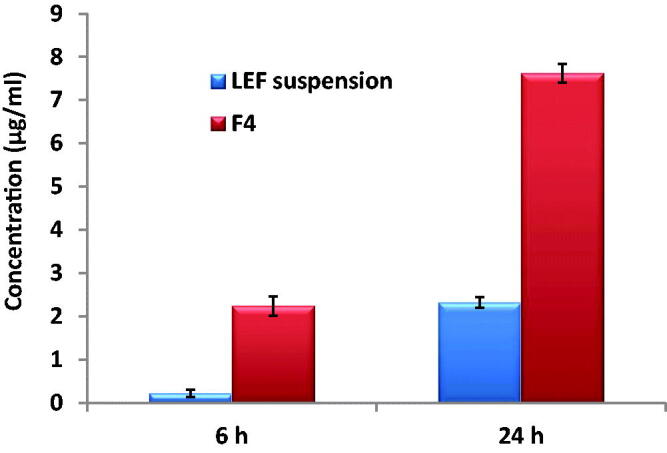
Uptake of LEF suspension and LEF loaded cubosomes (F4) by MDA-MB-231 cells after 6 and 24 h.

LEF uptake by MDA-MB-231 cells after 6 and 24 h was quantified by HPLC assay. As shown in Figure 8, for LEF suspension, LEF cell uptake after 6 hours was minimum and increased at 24 hours. LEF loaded cubosomes showed LEF cell uptake at 6 hours almost similar to the result of suspension at 24 hrs. Furthermore, LEF uptake again significantly increased in the cubosomal formulation at 24 hours. The higher cellular uptake of LEF from the cubosomal formulation in comparison to the free suspension is in accordance with the ex-vivo skin permeation results. LEF encapsulation in cubosomes improved drug permeability and consequently improved its activity as shown by its higher cytotoxic effect.

## Conclusion

LEF loaded cubosomes were successfully prepared using emulsification method and showed uniform cubic structure in TEM images. The prepared cubosomes had uniform particle size, high entrapment efficiency and they were able to provide LEF sustained release profile over 24 h. The selected formulation was stable for 3 months at 25 °C and had the highest percentage LEF permeated in ex-vivo skin study. In MDA-MB-231 cell line, LEF cubosome significantly reduced cell viability and showed higher cell uptake compared to LEF suspension indicating their potential application for breast cancer management.
